# e-Health Tools for Targeting and Improving Melanoma Screening: A Review

**DOI:** 10.1155/2012/437502

**Published:** 2012-12-13

**Authors:** Abhilasha Tyagi, Kimberly Miller, Myles Cockburn

**Affiliations:** ^1^Keck School of Medicine of USC, Los Angeles, CA 90089, USA; ^2^Department of Preventive Medicine, Keck School of Medicine of USC, Los Angeles, CA 90089, USA

## Abstract

The key to improved prognosis for melanoma is early detection and diagnosis, achieved by skin surveillance and secondary prevention (screening). However, adherence to screening guidelines is low, with population-based estimates of approximately 26% for physician-based skin cancer screening and 20–25% for skin self-examination. The recent proliferation of melanoma detection “e-Health” tools, digital resources that facilitate screening in patients often outside of the clinical setting, may offer new strategies to promote adherence and expand the proportion and range of individuals performing skin self-examination. The purpose of this paper is to catalog and categorize melanoma screening e-Health tools to aid in the determination of their efficacy and potential for adoption. The availability and accessibility of such tools, their costs, target audience, and, where possible, information on their efficacy, will be discussed with potential benefits and limitations considered. While e-Health tools targeting melanoma screening are widely available, little has been done to formally evaluate their efficacy and ability to aid in overcoming screening barriers. Future research needs to formally evaluate the potential role of e-Health tools in melanoma prevention.

## 1. Introduction

While the rates of many cancers are declining, melanoma rates are increasing worldwide [1]. In the United States, melanoma rates in white populations have been increasing by 2.4% annually [2]. Despite improved survival, mortality and morbidity remain significant for melanoma. Prevention efforts are thus critically important for this aggressive and often rapidly fatal disease [3]. 

Secondary prevention of melanoma is recommended as the disease is more easily treated and survival dramatically improved with early detection [4, 5]. Secondary prevention of melanoma is achieved by screening and skin surveillance performed by a clinician (dermatologist or other healthcare professional) or skin self-exam (SSE) performed by the individual. However, the value of population-based skin screening in melanoma prevention remains controversial. The US Preventive Services Task Force finds insufficient evidence from randomized controlled trials to recommend for or against whole-body skin examination by either clinicians or individuals [6], whereas observational studies have found evidence that both physician-based screening and SSE result in detection of thinner melanomas and, as a result, decreased mortality [7–9]. 

Despite these findings, and possibly due in part to conflicting guidelines, skin screening rates for melanoma remain low in the general population. According to data from the National Health Interview Survey (NHIS), physician-based screening rates are estimated at 16.5% in the US [10]. Skin self-examination estimates range between 20% and 25% [11]; however, these rates depend on the definition of SSE. When thoroughness or frequency of exam is included in variable measurement, SSE rates substantially decrease to as low as 7–9% [12, 13].

While early detection is key to improved melanoma survival in general, individuals in certain populations are at increased risk of poor outcomes as a result of advanced-stage diagnosis. In California Hispanics, rates of thick tumors whose prognosis is poor but that could be detected early with screening are increasing the most rapidly [14, 15]. Prevalence rates for skin examination, both physician-based and SSE, have been found to be lower in Hispanics and other minority populations [16, 17] as a lack of skin cancer awareness and poorer perception of risk may impede screening practices. 

To increase skin screening rates and broaden the range of individuals performing screening, strategies are needed that reduce adherence issues. A potential new approach to skin screening is in the proliferation of patient-oriented “e-Health tools,” digital resources that facilitate active self-management of patient health practices and information [18]. Low-cost and widely available e-Health technologies for skin surveillance may reduce practical and psychological barriers as they are patient-initiated, performed outside of clinical settings, and often personalized and interactive. With nearly half of the American adults using smartphones [19], and with 8 in 10 of the 82% of Americans who use the internet accessing health information online [20], e-Health tools for skin screening may more seamlessly match individuals' customary behaviors and open new channels for screening adherence. 

This paper provides a review of e-Health melanoma secondary prevention tools currently available as a reference source. In contextualizing and categorizing such tools, we aim to help focus formal research on their adoption and efficacy and to aid in determining which tools are most likely to provide effective ad hoc screening options in the future. We will examine how these screening tools work, how they are accessed, and whether and how they are validated. While detailed assessment of efficacy is beyond the scope of this paper, we will comment on the benefits and limitations of these tools and their potential for patient adoption.

## 2. Methods

We performed initial searches for Apple iPhone applications (apps) via the iTunes app store and Android apps via Amazon.com and Google Play app stores for the search term “melanoma.” We included mobile applications whose purpose is to increase user-dependent early diagnoses of melanoma. The means by which these mobile applications aid users in reaching diagnostic conclusions about moles include the use of mathematical algorithms to analyze skin lesions, education on skin self-examination or diagnostic criteria of melanomas, and photographic mole tracking and scheduled reminders to perform SSE.

We searched for additional e-Health tools via the Google Scholar database, Google search engine, and youtube.com. We also searched in the iTunes, Amazon.com, and Google Play app stores for apps that use similar means to increase early diagnoses of melanoma used by those apps discovered in the initial searches. The search terms we used included “melanoma,” “algorithm,” “video,” “mole,” “SSE,” “self-examination,” “interactive,” “mobile,” “e-Health,” “application,” “computer,” “online,” and “internet.” We further used the reference lists from articles to find additional e-Health tools.

We excluded e-Health tools that did not directly concern melanoma or that only gave informational material about melanoma rather than directly aiding in early diagnoses of melanoma, but included video-based tools that specifically aimed to educate in skin examination or diagnostic features of melanoma.

We summarized the available tools, including where and how to obtain them, their cost, and by and for whom they were developed, and, where possible, information on their accuracy and efficacy. 

## 3. Results

Table 1 provides an at-a-glance summary of the tools discovered in our search according to the means by which they improve early diagnoses of melanoma. Many of the tools use a combination of methods, but each is categorized by its principle means. For example, the My MoleChecker application has an embedded video demonstration on self-examination technique, but it is categorized as a mole tracking and reminder application because this is the primary method of melanoma screening for the application [60]. 

Searching for “melanoma” in the iTunes store for iPhone apps yielded 37 results, 17 of which were excluded based on the criteria described in Section 2. “Melanoma” was searched on the Google Play app store and yielded 29 results, 3 of which were also found in the iTunes searches and 21 of which were excluded according to exclusion criteria described in Section 2. A search for “melanoma” on the Amazon.com Android app store, produced 5 results—3 applications were discovered in other searches and 1 application was excluded the previously mentioned criteria. The 26 unique applications discovered by these searches are included in Table 1. 

Table 1 also includes those tools that were discovered in subsequent searches for tools in the same categories as these applications. Altogether, there are 46 e-Health tools summarized. 

### 3.1. Mobile Teledermoscopy

A dermoscope in a clinical setting magnifies a lesion to better elucidate its features beyond what is apparent by the naked eye for analysis by a clinician. Particularly in the past decade, teledermoscopy has been used to capture images of skin lesions to send to other clinicians for second opinions or diagnoses. Mobile teledermoscopy utilizes a dermoscope which attaches to the camera of a smart phone to capture standardized images. In a feasibility study, two teleconsultants evaluating images of skin lesions taken with the DermLite II PRO HR dermoscope were able to reach correct diagnoses on 91.5% of skin lesion images presented [67].

Several companies are making pocket dermoscopes, and while the devices are not currently targeted to consumers, pocket dermoscopes for smart phones capture images that yield high concordance diagnoses. Canfield, a well-regarded dermoscope manufacturer, produces the DermScope which fastens over an iPhone camera [23]. Canfield also produced a specific mobile application which serves to manage and store the images captured via that dermoscope. The dermoscope is targeted toward dermatology residents, primary care physicians who do not have access to dermatologists, and physicians who want to store and forward their images to a clinician who may have more expertise in making a diagnosis or suggesting a referral [23]. There are limited reports of patients performing dermoscopy as part of a self-exam [68]. 

### 3.2. Algorithm Analysis Apps

Algorithmic analysis e-Health tools work by analyzing the features of a user-provided picture of a suspect skin lesion and determining a risk profile for the lesion. The user typically manually inputs other information such as size and evolution of the lesion to further factor into the tool's risk assessment. Unlike the store-and-forward teledermatology devices in which a picture of a lesion is taken and sent to medical personnel for diagnosis, the algorithmic analyses provide diagnoses entirely without the oversight of a clinician. Although the particular algorithms vary from tool to tool, the evaluation of images approximate clinical criteria of melanoma such as asymmetry, border irregularity, color, diameter, and evolution of the skin lesion (ABCDE criteria) [29, 69]. For example, MelApp compares user-taken photographs to a database of skin lesion images to ultimately provide the user with a risk assessment of a particular mole [25]. The algorithmic tools are available via internet websites and smartphone applications. While the internet sites require an image to be uploaded with a separate camera, the smartphone applications are able to make use of the cameras built into the smartphone. The image quality, however, is limited by the quality of the camera used. The price for consumers ranges from free to a one-time fee for unlimited use to fee-per-use service. 

The SkinMD application developed by students at the University of Pennsylvania is free to download and allows users unlimited use of the application [31]. The majority of algorithmic programs available and advertised for consumer use have not yet undergone rigorous testing for validity. Those that have undergone testing for diagnostic accuracy have had specificities (benign detection accuracy) ranging from 65% to 93.8% and sensitivities (malignant detection accuracy) ranging from 71.1% to 100% [29]. 

MelaFind is a device intended for physician use that has premarket approval from the FDA [24]. Data from a multicenter prospective trial found that the sensitivity of MelaFind was 98.4% with a 95% lower confidence bound at 95.6% and biopsy ratio of 10.8 : 1 [24]. While arguably the Gold Standard for algorithm-based approaches, this application is not widely and readily available for the general public.

### 3.3. Demonstrative Videos

A number of e-Health apps include videos about melanoma and skin safety. Of interest here are those videos which include a demonstration of how to perform an SSE and how to identify suspect lesions and specific elements of melanoma prevention. Most videos that are produced for distribution to patients include not only information about SSE, but also incorporate skin safety advice, education on melanoma prevention, and motivational techniques to encourage frequent SSE. The “Skin Cancer: Learn to Spot it Early” video first includes facts about the risks of skin cancer and the benefits of early detection before demonstrating how to perform SSE [35]. 

Although not all of the videos shown to be efficacious in changing patient behaviors are available online, they could quite easily be uploaded onto a website for easy distribution to patients. For example, the videos from the “Check It Out” trial and Skin Awareness Study demonstrated efficacy in changing patient behaviors [36, 37]; these videos are not currently available to stream on the internet. Many health organizations produce videos, but the majority of the videos available online have not been assessed for their efficacy in changing viewer behaviors. For example, Saving Our Skin and Save Your Skin videos are easily accessible online for patients to access and use [38, 39], but there is no available data to confirm that these videos are utilized by patients at risk. SSE videos may be accessed through the websites of different organizations, on video websites such as youtube.com, and within mobile phone apps such as My MoleChecker [60]. Videos demonstrating SSE have been shown to increase viewer frequency of SSE in the short term [36, 37, 70–72]. There is limited evidence that videos are preferred to written materials particularly in older persons at risk for melanoma, rather than preferentially utilized by younger people who are less at risk, as has been assumed in the past [70].

### 3.4. Melanoma Educational Apps

Many websites and apps offer informational material about how to perform SSE. Generally these are expository tools without an interactive component that provide diagrams and text to explain how to perform a skin exam and how to recognize the features of a melanoma. The Mollie's Fund application provides information on SSE via a “5 Step Skin Check” as well as describing the ABCDE of moles using text and sample photographs in the app [41]. The Smack a Mole application offers images demonstrating the ABCDE of melanoma, as well as other general educational facts about skin cancer [46]. This particular application incorporates a game to draw the users' attention and keep them engaged.Although there are many of these types of educational e-Health tools, most have not been assessed for their efficacy. 

### 3.5. Interactive Teaching Apps

Interactive teaching methods range in their complexity, but involve the user performing some task such as answering quiz questions in order to progress through the tutorial. Skinsafe, an interactive app available for download via the University of Nottingham website, goes through various teaching modules and quizzes the user on recently presented information [54]. One of these modules entitled, “Spotting Melanoma,” explains how to discern moles from freckles and what warning signs may warrant a clinical consultation. This module finishes with a brief quiz which challenges the user to determine which moles in a series of photographs show warning signs of melanoma [54]. In a randomized trial, general practitioners and nurses were asked to prescribe Skinsafe to at-risk patients. Patients who received the Skinsafe intervention improved both skin cancer knowledge and screening behaviors, reporting more skin mole checks than did patients without Skinsafe at 6-month followup [73]. 

More sophisticated melanoma programs, such as eDerm, are targeted to educate medical personnel to better discern melanomas [51]. This program includes a lecture on skin cancers as well as benign skin lesions and rashes as well as interactive lessons with individualized feedback to increase the user's ability to distinguish skin lesions. Practitioners who completed the eDerm training program sent fewer of their patients to receive unnecessary biopsies [74]. 

### 3.6. Mole Tracking and Reminder Apps

Mole mapping software allows consumers to photograph moles and concurrently save the date and time of the mole for future comparison of “evolution” (the “E” in ABCDE). Mole tracking applications are usually paired with a reminder feature to remind the user to perform another skin exam at a certain time in the future, in order to track the evolution of the mole. The UMSkinCheck application guides users through full-body skin surveys, tracking specific lesion, and allows users to set up self-exam frequencies [55]. In addition, UMSkinCheck provides information on the ABCDE of melanoma, common skin lesions, and sun protection [55]. Similarly, the Track-A-Mole application allows users to set alarms for subsequent skin examinations [57]. Mole tracking software is largely available via mobile applications, which utilizes the camera hardware already in the phone. While mole tracking and reminder applications have not been studied for their effectiveness in particular, reminder functions via mobile phones have been shown to increase sun-safe behaviors such as to apply sunscreen and wear sun-protective clothing [75]. 

## 4. Discussion

In the past several years, there have been many advances in e-Health that have potential to improve melanoma screening: all of the mobile applications and the majority of the e-Health tools cataloged in this paper were released within the past four years. 

The central appeal of e-Health devices in the context of melanoma is to increase patients' involvement in their own healthcare and ultimately change patients' behaviors so that melanomas can be found at earlier stages when treating with curative intent is possible. e-Health may achieve these ends through various means including serving as diagnostic devices, teaching skin self-exams (SSE) through demonstration, explaining the ABCDE diagnostic features of melanoma through interactive teaching or exposition, and by offering mole tracking and SSE date reminders to assist users in regularly evaluating their moles. 

By encouraging greater and more autonomous involvement in health care, the use of e-Health tools offers opportunities to provide more comprehensive patient care and bolster the relationships between patients and clinicians [37]. e-Health tools for screening might resolve the gap for primary care physicians as well, providing easily accessible training on performing clinical skin exams [71] (e.g., by such websites as eDerm) [72]. In a survey of primary care physicians (PCPs), nearly half of the respondents reported that they did not perform skin exams and that a common barrier to performing exams was a lack of confidence in identifying skin lesions [41]. e-Health devices may address these issues from the physician's side as they may encourage greater screening and referral in the primary care setting. 

The increasing availability of e-Health devices might also benefit patients in remote locations without access to specialists, or patients with physical barriers that affect access to care. With an increasingly aging population and limited number of health facilities, e-Health technologies might be particularly useful in elderly populations that tend to be more isolated [46]. While older adults use technology less [54, 73], studies have indicated that elderly patients trained to use certain devices found the technology easy to use and demonstrated a high level of satisfaction with the delivery of e-Health services [51, 55, 74]. Thus there is potential for such tools to improve screening rates in this at-risk population [57].

Conversely, the use of e-Health devices may potentially disrupt collaborative relationships between patients and physicians [75] as information is presented within a limited context which may lead to miscomprehension of medical diagnoses. e-Health tools may be used in lieu of clinical visits, and patients may overestimate their accuracy. Such overconfidence might lead patients to neglect a physician's consult that may have been essential to discovering a melanoma at a treatable stage [21, 22, 26].

Thus, while e-Health tools have tremendous potential to diagnose melanomas at an earlier stage, we must also consider the potential harms of e-Health tools to screen for melanoma that derive from the limited oversight of their use by professionals. As is the case with any screening approach, we should consider the potential harms of e-Health as a screening method, as well as the potential benefits, and this should be done in a formal and systematic manner.

### 4.1. Envisioning e-Health Device Use

A significant barrier to widespread promotion of existing e-Health tools is an absence of efficacy testing. Increasing the independent testing of e-Health tools can aid in determining which tools may have life-saving benefits to individuals and eliminating other tools that may potentially harm users. A study comparing the efficacy and accuracy of e-Health devices with similar purposes may be beneficial to determine whether health care providers and organizations ought to endorse those devices. Such a study might also elucidate user preferences for certain tools based on characteristics including user engagement and ease-of-use. 

It is important to learn whether those who access direct-to-consumer e-Health devices are the same as the target population for those devices. It is possible that the individuals who discover e-Health tools likely have preexisting knowledge of the tools or are particularly motivated to seek such tools. Such users may already be highly involved in their own health care and although eager to use e-Health tools, they do not receive any additional benefit from them. If this is the case, studies should be performed on how to best market devices to particular high-risk target populations. 

Physicians must also have prior knowledge of e-Health devices in order to recommend the devices to patients. Educating a collection of physicians who would be able to prescribe e-Health tools to patients with certain risk factors may be an efficient means by which patients can become aware of useful devices. 

## 5. Conclusions

Melanoma screening is required in order to reduce morbidity and mortality from a disease whose incidence is increasing, and for which little advancement in treatment modalities has been achieved. There are many available approaches to melanoma screening, but all share a common drawback that levels of uptake and screening adoption are too low for even an accurate screening method to effectively reduce mortality. The availability of e-Health tools to a large proportion of the population with access to mobile devices offers one avenue for improving adoption and uptake of simple screening tools. However, as is the case with any screening method, careful analysis of the accuracy and efficacy of each of these tools is required, and that evaluation should include analysis of the potential drawbacks to their use, such as generating a false sense of security with a false negative outcome. 

While e-Health applications offer much promise for the improvement of melanoma screening, current production and evaluation efforts are *ad hoc*, and most apps have some, but not all, required features. Very few have been formally tested, and doing so could be an excellent step forward in widening the scope of effective melanoma screening.

## Figures and Tables

**Table 1:** e-Health tools for early diagnosis of melanoma.

Device	Accessible via	Target	Developer	Cost
Mobile teledermoscopy				
Handyscope [21]	http://handyscope.net/	Physicians	FotoFinder Systems GmbH	$609.59^1^
DermLite II PRO HR [22]	http://www.dermlite.com/cms/	Physicians	3Gen LLC	$1,295.00
DermScope [23]	http://www.dermscope.com/	Physicians	Canfield	$895.00

Algorithm analysis apps				
MelaFind [24]	http://www.melafind.com/	Physicians	MELA Sciences Inc.	$7500
MelApp [25]	Apple app store	Consumers	Health Discovery Corp.	$1.99
Mole Detective [26]	Apple and Android app stores	Consumers	New Consumer Solutions LLC	$4.99
Doctor Mole [27]	Android app store	Consumers	Mark Shippen	Free
Moletest [28]	http://www.moletestuk.com/	Consumers	Moletestuk	$31.28^2^
“Internet-based” [29]	http://dermoscopy.k.hosei.ac.jp/	Consumers	Iyatomi et al.	Free
Skin Scan [30]	Apple app store	Consumers	Skin Scan	$4.99
SkinMD [31]	Apple app store	Consumers	University of Pennsylvania students	Free
Skin Of Mine [32]	Apple app store	Consumers	Medical Image Mining Laboratories, LLC	Free
SpotMole [33]	Android app store	Consumers	Cristian Munteanu	Free
SpotMolePlus [34]	Android app store	Consumers	Cristian Munteanu	$1.53–1.64^3^

Demonstrative videos				
“Skin Cancer: Learn to Spot it Early” [35]	http://azcc.arizona.edu/	Cutaneous oncology patients	Arizona Cancer Center Skin Cancer Inst.^4^	Free
“Check it Out” [36]	(not online)	Primary care patients	American Cancer Society	Free
Skin Awareness Study [37]	(not online)	High risk patients	Janda, et al.	Free
Saving Our Skin [38]	http://www.youtube.com/	Consumers	Cancer Council Queensland	Free
Save Your Skin [39]	http://preventcancer.org/	Consumers	Prevent Cancer Foundation	Free
Skin Self-Exam [40]	http://thedoctorstv.com/	Consumers	2012 STAGE 29, LLC.	Free

Melanoma educational apps				
Mollie's Fund [41]	Apple and Android app Stores	Consumers	MCS Advertising	Free
iSkin [42]	Apple app store	Consumers	DKLO Inc.	$0.99
Melanoma Watch [43]	Apple app store	Consumers	Stroika	Free
Mole Checker [44]	Apple app store	Consumers	Stroika	$2.99
Mole Checker [45]	Android app store	Consumers	Harry Arden	$2.49
Smack a Mole [46]	Apple app store	Consumers	SilkyDragon	$2.99
ABCDEs of Melanoma [47]	Android app store	Consumers	Mouhammad Aouthmany	Free
MelanomaExposed [48]	http://www.melanomaexposed.com/	Consumers	Bristol Myers Squibb	Free
Cancer Council Queensland [49]	http://cancerqld.org.au/	Consumers	Cancer Council Queensland	Free
My Self Checker [50]	Apple app store	Consumers	Channel 4	Free

Interactive teaching apps				
eDerm [51]	http://www.westportal.com/prodederm.htm	Physicians, students	West Portal Software Corp.	Free
Melanoma Visual Risk Calculator [52]	Apple app store	Consumers	SigveDhondup Holmen MD	Free
SPOT IT & NAME IT [53]	Apple app store	Consumers	ECD-Network, LLC	$2.99
Skinsafe [54]	http://nottingham.ac.uk/	Primary care patients	University of Nottingham	Free
Mole tracking and reminder apps				
UMSkinCheck [55]	Apple app store	Consumers	University of Michigan	Free
MoleTrac [56]	Apple app store	Consumers	Depthmine Software	$0.99
Track-A-Mole [57]	Apple app store	Consumers	Peak Mobile Designs	Free
Skin Prevention [58]	Apple app store	Consumers	DIMENSION S.r.l.	$6.99
LoveMySkin [59]	Apple app store	Consumers	Steven Romej	$0.99
My MoleChecker [60]	Apple app store	Consumers	Channel 4	Free
Skin Scanner [61]	Apple app store	Consumers	Intelligent Life Solutions	$0.99
SkinTagger [62]	Apple app store	Consumers	Coriumedic Systems LLC	Free
SkinKeeper [63]	Apple app store	Consumers	Health Safari Pty Ltd.	$3.99
nēvus [64]	Apple and Android app stores	Consumers	Shonik IDEAS	$2.99
Mole Measure [65]	Apple app store	Consumers	Robert Dewhurst	$4.99
YourSkinDiary [66]	Apple app store	Consumers	Buiss Ultimo	Free

^1^Conversion of 495.80€ price.

^2^Conversion of *£*19.95 price.

^3^$1.53 price quoted from the Amazon.com App Store and $1.64 price quoted from Google Play App Store.

^4^Collaborative effort of the Arizona Cancer Center Skin Cancer Institute, the College of Nursing and the Office of Instruction and Assessment (OIA).
